# Chronic Nasal Catarrh

**Published:** 1884-07

**Authors:** R. O. Carter

**Affiliations:** Atlanta, Ga.; Assistant to Chair of Eye, Ear and Throat Diseases, Atlanta Medical College


### CHRONIC NASAL CATARRH.*

BY R. O. CARTER, M. D.,

Assistant to Chair of Eye, Ear and Throat Diseases, Atlanta Medical College.

Chronic nasal catarrh is an inflammation of the nasal mucous membrane, causing a chronic condition of impediment to nasal respiration, accompanied with more or less mucous, or muco-purulent discharge. This inflammation may, and very frequently does, extend to the conjunctiva by way of the nasal duct, and may involve the frontal and ethmoid sinuses, and also the antrum of highmore. It is usually accompanied with hypertrophic thickening of the mucous membrane covering the turbinated bones, especially the lower ones. It often begins as simply an idiopathic acute coryza, repeated attacks of common cold neglected, perhaps, and improper hygienic precautions against the same, causing the disease to extend into chronic nasal catarrh. In other cases it appears so gradually as to seem chronic, as it were, from the beginning. In many cases the variety which I shall designate as nasal or anterior nasal catarrh, is plainly hereditary. In this variety I shall also class syphilitic nasal catarrh, as syphilitic and scrofulous nasal-catarrhs generally manifest themselves in this form, rather than in the variety which I will call post nasal, or naso-pharyngeal catarrh.

My observation is, that post nasal catarrh is much more frequently met with, at least in this section, than any other variety. It is most frequent generally in damp situations where people are frequently subjected to changes from warm dry rooms to a cold damp atmosphere outside. It seems that our climate of Atlanta, while it is certainly a most admirable one during most of the year, is during the months of January, February and March, when the rainy and muddy season prevails, specially prone to the development of nasal

*Read at a meeting of the Atlanta Medical and Surgical Union.

catarrh. Indeed, I think it advisable for practitioners here, when practicable, to send such patients to other dry and milder climates—say Florida—during these months, as I firmly believe that, for the patient to remain in Atlanta during these months, will retard his cure very considerably. Exposure to draughts, and sitting with damp clothing or wet shoes on, is a very common cause of the disease, and one which certainly aggravates an already existing catarrh As to tobacco smoking, and especially cigarettes, I am absolutely certain that this is a factor in the production of nasal catarrh, which, from the slight importance attached to it by our writers on this subject, is not generally recognized. I am positive that it is a very frequent cause, and that bad cigars and tobacco will naturally be more prompt in causing the disease than a better quality will. Of course, exposure in dusty localities and cold, raw winds, are also frequent causes of the disease. As the symptoms of the disease are very well known, I shall be as brief as possible in the relation of them. I shall also have to omit, for want of space, many of the important complications of the disease. In post-nasal catarrh, the patient will generally, not until the disease has been in progress for some weeks or months, present himself for treatment. He is constantly hawking and spitting a frothy mucus, very much like thin starch as we see it prepared for laundry use. He will also be constantly blowing his nose and will saturate one-half dozen to a dozen handkerchiefs during the day. As each effort to blow his nose causes a congestion of the sponge-like schneiderian membrane there will frequently be tinges of blood in the discharge, so much so as to often render the handkerchief quite bloody. The nasal mucous membrane will be so thoroughly infiltrated with serum that air cannot pass through without great difficulty, and this especially happens when the patient is lying in bed at night, the serum falling by gravitation into the sponge-like mucous membrane of the nostril upon which he is lying. This stuffy sensation about the nasal passages and naso pharynx is very annoying and harassing to the patient. He has to lie with his mouth open in order to breathe, and this constant exposure of the pharynx to currents of cold air, brings on more or less chronic pharyngitis and hypertrophy of the mucous membrane of the larynx, etc., with hoarseness and chronic throat disease, and if the patient has to earn his living by singing or public speaking, his occupation is gone, at least until his disease is cured. To resume, the patient blows his nose, and the effort to clear it only increases the congestion and difficulty, it seems. The profuse mucous discharge drifts down behind the soft palate, and he either has to swallow it—and the diseased secretion thus swallowed produces many a case of dyspepsia,—or he lies awake, hawking and spitting, is deprived of his sleep night after night, and thus is laid the foundation for chronic insomnia, which is far more detrimental than his catarrh. These patients are specially prone to suffer from melancholia and mental depression. The constant hawking and blowing renders them unfit to enjoy society. The disease generally implicates the nasal duct, and may go on and produce stricture of the same, and require an operation for its relief. One of the most annoying and very frequent complications is the catarrhal conjunctivitis, of varying degrees of intensity, which it produces. This is really about as annoying to the patient who has to use his eyes a great deal, as the catarrh itself. The hypertrophic condition of the naso-pharynx is very annoying to especially those persons who have to use their voices a great deal, as it interferes so seriously with phonation and pronunciation. It will also extend to the larynx and the mucous membrane about the upper and lower vocal cords becomes more or less red and congested. The mucous glands of this region now secrete a profuse and abnormally thick secretion. Singers and public speakers are greatly annoyed thereby, and the shortest effort at using their voices soon tires their throats. For the palliation of this most annoying symptom, I have, along with the general treatment of the disease, often been successful in the use of compound eucalyptus lozenges, and lozenges of muriate of ammonia and chlorate of potash. Also by the inhalation of carbolic acid and tinct. of iodine; in equal parts, dropped on a sponge placed in an empty morphine bottle, this to be inhaled slowly several times a day. These are very useful in aiding to relieve the hoarseness. A gargle also of warm salt water is very good. I have seen several cases in which the disease extended to the frontal sinuses, causing severe inflammation and great pain; so much so as to nearly derange the patient's mental faculties, and finally the pus would burrow through the necrosed wall of the orbit or frontal sinus, and establish a steady flow, requiring months of the most careful treatment to cure. By way of parenthesis, I will say of the cases of this complication which I have treated—I always had to make a counter-opening by way of the nostril, if one was not already formed, in the carious bones of the nose. This is usually easily done. You thus get an exit through the inside of the nostril for your injections. I treated my cases by injecting through a long, slender, pointed syringe, and in some cases, I had to enlarge the fistulous opening so as to be able to introduce my syringe. I used generally one-half to 4 gr. to the oz., sol. of chlo. zinc, 2 to 5 gr. plumbi acetat. sulph. zinc, etc., and various strengths of carbolic acid and labarraques solution, repeated every day at first and gradually increasing the intervals. After the syringing I packed the fistula with lint. I consider it also very necessary to give iodide potash as an alterative in such cases and to assist in the elimination of the diseased bone, no matter whether there is any syphilitic or scrofulous complication or not.

In post-nasal catarrh, the sense of smell is often obtunded to quite a considerable degree, but this generally prevails only during acute exacerbations of the disease, and so far as I have observed, this symptom is nothing like so serious as it is in anterior nasal catarrh and ozena. In the post-nasal form of catarrh the bones of the nose, etc., are not attacked, nor is the mucous membrane ulcerated, as it is liable to become in anterior and syphilitic catarrh. Of the pharyngeal part of the disease, I am sorry I have not space to say more, as it is such a very important feature of it. In this condition the patient complains, in addition to the forementioned symptoms, of a burning, stinging pain, or a hot, dry, stuffy sensation above the palate, with perhaps, the usual profuse mucous discharge. Examination with the rhinoscopic mirror will show the glands on the superior surface of the palate and the roof of the naso-pharynx, and especially around the pharyngeal mouth of the eustachian tube, enlarged and profusely secreting a diseased mucus. This, of course, accounts for the many cases of aural catarrh we have to treat, which have their origin in this way. The disease extending by continuity of tissue to the middle ear, causing hypertrophy, and in some cases finally atrophy of the mucous lining of the eustachian tube and tympanum, with tinnitus aurium, etc., and finally, if not checked by proper treatment, more or less deafness.

Examination of the anterior nares by means of the speculum, (and I prefer the bivalve speculum), will generally show an abnormal redness of the nasal mucous membrane, and a bulging inwards of the mucous membrane of especially the lower turbinated bones. This often so resembles polypi as to have frequently been mistaken for such.

Rhinoscopic examination will also show about the same condition at the posterior nares. The vomer usually impinges on the left side, and this side of the nose will be the one most usually occluded.

Among the nervous symptoms noticed in post-nasal, as well as anterior nasal catarrh, are most painful and annoying frontal and occipital neuralgias, together with loss of memory and lack of power of thinking for any considerable length of time upon any one subject. This latter symptom, I think, may be accounted for by the nervous worry which the disease causes. This also causes many a case of nervous dyspepsia, while the odor, when there is any, in a case of post-nasal catarrh, is not usually sufficient to amount to much notice, there is sometimes a peculiar odor, to which Dr. Calhoun has often called my attention. It very much resembles that of semen more than anything else, and is most typical in old persons, and especially in old women. As to the prognosis of post-nasal catarrh, I will simply say, my experience is that many cases can be cured, and all can benefited by proper treatment, if kept up a sufficient length of time.

ANTERIOR NASAL CATARRH AND OZENA.

This variety is usually, I think, a separate disease from post-nasal catarrh, and yet the two often co-exist. In anterior nasal catarrh the discharge is generally quite profuse and yellow, and often has a most sickening and offensive odor, so much so as to render proximity to the patient very obnoxious, though the patient's sense of smell is often so obtunded he himself may not be aware of it. The matter often hardens and forms into scabs and slugs as large as the finger-nail, or may be an inch long or more. These may be very difficult to dislodge, and are perhaps finally blown out at the anterior nares, or hawked back and spit out. Syphilitic catarrh is generally the worst form of anterior nasal catarrh, owing to the destruction of the hard and soft tissue which it causes. The nasal mucous membrane is not often ulcerated, except in the syphilitic or scrofulous variety; and yet it is by no means correct to say that ulceration or necrosed bone in every case of nasal catarrh is necessarily syphilitic. In bad forms of anterior nasal catarrh the nasal septum is frequently perforated. Cases are often seen in which the end of a finger can be passed through the hole in the septum, and the turbinated and nasal bones are liable to become necrosed, and work out by piecemeal. These spiculæ of bone will often require a great deal of trouble and skill in their removal, but must be gotten rid of, either by the douche, or by proper manipulation with forceps, etc. The term ozena is applied to the disease when in this offensive or destructive form, when the bones especially are attacked. Great deformity, of course, is apt to ensue upon destruction of the nasal bones. In this form the disease often extends to the frontal and ethmoid sinuses and the autrum of highmore, and may even, in rare cases, attack the meninges of the brain, and thus terminate the disease, together with the life of the patient. The sense of taste, though more especially that of smell I should say, is frequently greatly obtunded. The prominent causes of this variety of catarrh, aside from those mentioned in speaking of post-nasal catarrh, are hereditary, and I mean without any strumous or syphilitic taint, injuries of the nasal bones, etc., very often syphilis and also scrofula. It is of course a much more formidable disease than the post-nasal variety. As to prognosis, while it is not so favorable, yet I think it is entirely correct to say that many cases can be cured, and all can be benefited. It will, of course, not do to let it go untreated.

TREATMENT.

In speaking of the treatment of catarrh, I will endeavor to be as brief as possible, stating in the beginning that the plan of treatment which I shall detail is, in the main, about what is generally adopted by the best known and most successful specialists who treat these diseases.

I will begin by giving the plan of treatment I generally adopt in a typical case of post-nasal catarrh, and will speak only of those remedies which I know to be good. First, the patient must have a Warner's post-nasal douche, which he can readily procure at a drug store at a very small expense (75c. to $1.00). The value of this little instrument is almost inestimable. It is to the ear and throat specialist what the Sims speculum is to the gynœcologist, invaluable. Cold water should never be used in the douche, as it not only causes severe pain to the nostrils, but may get into the eustachian tubes, and cause an acute inflammation of the middle ear. Let the patient use a drachm or more of common salt to a cupful of warm water. This will be about three or four douchefuls, and is enough in an ordinary case of post-nasal catarrh. There are directions accompanying the Warner's p. n. douche, and the patient soon learns to use it properly. He should stand before a mirror in front of a good window light, and insert the nozzle of the douche carefully up behind the soft palate so as to avoid coughing and gagging, and with the head bent forward, but not so low as to favor the flow of the stream into the nasal duct; he should rather slowly but firmly squeeze the rubber bag and wash out the nostrils through the anterior nares. In a severe case this should be done twice a day, and in milder cases once a day. Now, after washing with the salt water, there may be frequently a feeling of stuffiness in the nostrils, and a desire to blow the nose, but after a few minutes this generally gives way to a grateful sense of coolness and relief. After the salt water douche I generally employ some one of the following washes:

℞. Mur. ammonia, ℥ss., aq. dist., ℥viii. M. and Ft. sol. sig.; use 1 to 2 teaspoonfuls to the douche; when I say douche I mean one douching, that is one cupful of warm water.

℞. Tanno glycerine, ℥viii.; sig., use ½ to 1 teaspoonful to the douche; though I am rather beginning to lose confidence in tanno-glycerine.

℞. salicylic acid, sig. Begin with a small pinch and gradually increase to what strength the patient can bear the solution, and if at first it burns, add a little bicarbonate of soda.

℞. Labarraques sol. Begin on 5 drops to the douche and gradually increase to 1 teaspoonful. If there exists a great deal of hypertrophy of the schneiderian membrane, and especially of the lower turbinated bones, I think highly of chloride of zinc, though it is a very severe astringent to the nasal mucous membrane. Yet it has in my hands been very satisfactory in assisting to reduce this hypertrophic condition. I usually begin on ½ gr. to the ℥, and even this strength has caused, in some of my cases, the most distressing frontal neuralgias. I have, in some cases, gradually brought it up to 4 or 5 grs to the ℥.

I have used the following often in post nasal catarrh, though I generally prefer it in cases of ant. nasal catarrh where the patient has to use a larger quantity, and it is so cheap, yet very good indeed:

℞. Ferri sulph., ʒi.; Cupri. sulph., ʒi. M. and Ft. sol., sig. use. Aq., ℥ii., in douche; begin on five drops and gradually increase to 60.

These are about all the washes I use in post nasal catarrh, and they should be alternated every week or so. Probably the salt water is about as efficient as all the rest put together in a mild case of catarrh. I am almost prepared to believe so. I also use the following ointment of iodoform to aid in the reduction of the hypertrophy, and also for its general effect upon the disease, and I think highly of it:

℞. Iodoform, pulv., ʒi., ext. geranium, gr. x., acid carbol., gr. xv., vaseline, ℥i. M. and Ft. ungt. sig.; apply to nostrils at night. This may be applied by either the camel's hair brush, or by saturating a small pledget of absorbent cotton with the ointment and packing it in one nostril, and alternating each night between the two nostrils. It is claimed that the addition of 20 gr. chloral hydrate added to the above formula will deodorize it to some extent, and I often employ it. Some practitioners attach a good deal of importance to the insufflation and application of various powders, astringent and otherwise, and a snuff of equal parts of white sugar and camphor is frequently used. I am sorry to say that I have not been able to get any good results from any of the powders, etc., which I have used. On the other hand, they only caused a troublesome sneezing, and increased the congestion and aided in the blocking up of the nasal passages. In some cases, after the most of the annoying symptoms such as the discharge, sneezing, etc., have passed off, there will remain a hypertrophic condition of the mucous membrane which has to be removed, if at all, by surgical means. Excision by means of the wire snare is a very popular way of doing this now, and while I have not used this, I have every reason to think highly of it. Grasping the polypus-like membrane with toothed forceps, and clipping off a piece with the scissors, and afterwards applying lightly the solid stick of nitrate of silver, and the resulting contraction of the cicatrix, is another valuable means of relieving this condition, which I have seen employed.

In anterior nasal catarrh we adopt about the same measures as we have above described in post-nasal, and yet none is generally necessary. In this form we will often have to use the anterior douche, in addition to the post-nasal douche. The anterior douche is, of course, well known, and its object is to throw a jet of water into one nostril and carry it out at the other side. A very serious objection to the anterior douche is that there is a liability of throwing irritating solutions into the eustachian tubes, owing to their direction, and causing inflammation of the middle ear; and yet I have in two cases caused this same trouble with the post-nasal douche, when very strong solutions were used.

It has been suggested by Cohen, I believe, and very sensibly too, that this danger may be obviated when using any douche, by simply refraining from the act of swallowing, as occlusion of the pharyngeal mouth of the eustachian tube is maintained by the opposition against it of the contracted levator muscle of the palate, and the relaxation of this muscle of course exposes the mouth of the tube, and besides, during the act of swallowing, the air in the naso-pharynx is condensed and naturally rushes into the tubes and of course the solution tends to go with it. Now I am satisfied from experience, that such harmless solutions as warm salt water, which often rush up into the eustachian tubes and are plainly felt by the patient, instead of doing harm, are rather beneficial in those cases of catarrh where there is such a frequent tendency to aural catarrh. Still we must try and guard against the introduction of irritating solutions into the middle ear, and if I find in any case the anterior douche causes it, I stop its use and employ the posterior douche. So in anterior nasal catarrh we use every day, once or twice daily, the forementioned solutions, as spoken of in post-nasal catarrh. If we use the anterior douche, we must, of course, use more water, and the solution can also be borne stronger; we here use one pint to one quart of water at each sitting. We will generally find it necessary to use iod. pot. as an alterative, no matter whether there are any syphilitic complications or not, and of course if syphilitic, we must use proper anti-syphilitic measures, and in any case, if necessary, build up the patient by using good tonics, such as Iron, Strychina, Quinine, phosphorus, etc., and by all means cod liver oil in winter, and if they cannot take cod liver oil, as they frequently cannot as the weather grows warmer, I consider the Fellow's syrup of hypophosphites about the best substitute I have used. The treatment of both anterior and posterior nasal catarrh must be kept up from two or three months to two or three years. I do not mean that the treatment should be kept up regularly all this time, but if the case progresses favorably, after the first month or so, the douche may be used, say every few days Patients will often get disgusted and tired of the treatment, and quit it from time to time, and it will tax your ingenuity and skill to impress upon them the importance of keeping it up.

Now, I have reserved some of the most important points in the treatment to the last of this paper. I am greatly impressed with the belief that our writers upon this disease generally say too little about hygiene in the treatment of it. Habitual constipation, besides being a form of indigestion, undoubtedly leads to a congestion of the schneiderian, and naso-pharyngeal mucous membrane, and thereby retards the case. Then this must be corrected, and there is no surer way of doing it than by forming the habit of emptying the rectum at a certain time every day. If the digestion is out of order how quickly we notice that acute exacerbations of catarrh, and of catarrhal neuralgia are set up. It will show itself in a very few moments after partaking of some indigestible article of food or too hearty a meal. So we must carefully guard against indigestion by all the means in our power. If the patient is dyspeptic he must shun coffee as he would a poison, for I am sure that coffee is almost an irritant poison to a weak or irritable stomach. As to clothing, warm flannels must be worn next to the skin in cold weather, and by all means good strong, thick-bottomed shoes in cold and damp weather, and when the patient comes into the house with wet shoes on, he should, by all means, instantly exchange them for a dry pair. This I consider entirely practicable, and is, I am sure, most important, and the neglect of it causes the retardation of a cure of many a case of catarrh. If rubber overshoes are worn, they should be removed immediately upon coming into the house, as they retain perspiration and moisture, which dampens the feet. As to tobacco smoking, there is no other thing which has been so conclusively demonstrated to my experience as its evil effect upon catarrh. If your patient persists in smoking, you had about as well tell him to seek medical advice elsewhere. Not only must he not smoke himself, but he should not remain in a room, if he can avoid it, where others are smoking, as the irritating effect of the smoke thus inhaled is about as bad. Some writers advise the smoking of cubeb cigarettes to relieve the stuffy sensation in the nostrils. Possibly this may have some tendency to do this for a few minutes, but my experience is, that they do harm, and finally increase the very trouble for which they are used, and I now advise against their use. I will also state that while anterior nasal catarrh may and often does extend backward, and become complicated with post-nasal, the post-nasal form of catarrh is by no means apt to be converted into anterior catarrh, unless perhaps, the patient should contract syphilis. I have not had sufficient space to hardly allude to many of the prominent complicatins, or seqaelæ of nasal catarrh, such as aural catarrh, naso-pharyngitis, etc. These two especially are of such importance as to demand separate consideration.

